# Impact of nutritional profile on pain and functionality in patients with frozen shoulder: a cross-sectional observational study

**DOI:** 10.3389/fmed.2026.1785577

**Published:** 2026-04-15

**Authors:** Dina Hamed-Hamed, Oscar Daniel Rangel-Huerta, Eduardo Casas-Albertos, Celia Rodríguez-Pérez, Santiago Navarro-Ledesma

**Affiliations:** 1 ClinicalMedicine and Public Health PhD Program, Faculty of Health Sciences, University of Granada, Granada, Spain; 2 Hospital Universitario de Melilla, Melilla, Spain; 3 SpectraMinds, Nordre Follo, Norway; 4 Department of Nutrition and Food Science, Faculty of Pharmacy, University of Granada, Granada, Spain; 5 UGR’s “José Mataix” Institute of Nutrition and Food Technology (INYTA), Biomedical Research Centre, University of Granada, Granada, Spain; 6 Instituto de Investigación Biosanitaria ibs.GRANADA, Granada, Spain; 7 Department of Physiotherapy, University of Granada, Granada, Spain; 8 Faculty of Health Sciences, Research Unit of Excellence of the Melilla University Campus (UECUMEL), Melilla, Spain

**Keywords:** chronic low-grade inflammation, frozen shoulder, immunometabolic mechanisms, micronutrients, nutritional profile

## Abstract

**Introduction:**

Frozen shoulder (FS) is a chronic and painful joint disorder characterized by progressive stiffness and functional limitation. Its multifactorial pathophysiology remains poorly understood, although emerging evidence suggests a role for low-grade inflammation, metabolic dysregulation, and immune–endocrine imbalance. Nutrition and lifestyle factors may therefore represent modifiable contributors to disease development and progression.

**Methods:**

This cross-sectional observational study investigated the association between dietary profile, physical activity, pain intensity, and shoulder function in 57 patients with FS (44 women and 13 men). Sociodemographic variables, physical activity levels, adherence to the Mediterranean diet, detailed dietary intake, and shoulder pain and disability were assessed using validated instruments. Multivariate analyses, including Elastic Net and sparse partial least squares regression, were used to identify dietary and lifestyle predictors of pain and functional impairment.

**Results:**

Higher consumption of refined carbohydrates, starch, total cholesterol, and processed animal products was associated with worse shoulder function. In contrast, greater intake of specific micronutrients, including thiamine, niacin, iron, manganese, and vitamin D, as well as decaffeinated coffee and higher physical activity levels, was associated with lower pain and disability, particularly in women. Sex-stratified analyses showed stronger and more consistent associations between micronutrient intake and clinical outcomes in women, while predictive modeling in men was limited by sample size.

**Discussion:**

These findings suggest the presence of a nutritional pattern associated with symptom severity in FS, supporting a potential immunometabolic contribution to the condition. However, due to the cross-sectional design, causal relationships cannot be established and reverse causation remains possible. These results should be interpreted as hypothesis-generating and highlight the need for longitudinal and interventional studies to determine whether dietary and lifestyle modifications can influence clinical outcomes.

## 1 Introduction

Frozen shoulder (FS), is a frequent condition that affects approximately 2–5% of the general population, with higher prevalence among individuals between 40 and 60 years of age (1). This condition is characterized by a painful and gradual loss of active and passive shoulder movement, due to fibrotic proliferation of tissue accompanied by inflammation, neoangiogenesis, and neoinnervation (2), as well as contracture of the shoulder joint capsule (3). On average, the duration of this condition is around 30 months, although in some cases it may be significantly prolonged, representing a major burden for both affected individuals and healthcare systems (4).

Although the term “frozen shoulder” was introduced by Codman in 1934, who described it as a disorder that is difficult to define, treat, and explain (4), this term remains relevant today due to the lack of understanding of the molecular mechanisms responsible for the underlying inflammation and fibrosis of the glenohumeral capsule (5). Microscopically, the affected capsule shows an increased number of fibroblasts, mast cells, macrophages, and T lymphocytes (6). This synovitis is associated with elevated levels of fibrotic growth factors, inflammatory cytokines, and interleukins. In turn, this leads to an imbalance in extracellular matrix turnover and a thickened, rigid glenohumeral capsule with an abundance of type III collagen (7). Furthermore, a state of low-grade inflammation associated with diabetes, cardiovascular disease, and thyroid disorders appears to predispose individuals to the development of FS (7).

Pain and restricted mobility are the most common symptoms affecting shoulder function in daily life activities and quality of life (8). FS has been categorized into primary and secondary forms (9). The primary variant is characterized by a gradual onset with no known cause, while secondary FS is associated with a specific event such as a rotator cuff injury or trauma (9). This condition has been described to progress through three transitional phases: increasing pain and progressive stiffness, persistent stiffness and decreased pain, and a resolution phase in which residual pain diminishes and movement improves (10).

The diagnosis of FS is primarily based on clinical findings (2), although some studies support early detection of changes through magnetic resonance imaging (MRI), such as thickening of the coracohumeral ligament, axillary recess, and capsule within the rotator interval (11).

There is currently no definitive treatment for this condition, and consensus on optimal evidence-based management is limited (12). Current treatment options include physical therapy, corticosteroid injections, mobilization, capsulotomy, and manual rehabilitation therapy (13). However, the effectiveness of these treatments remains insufficient (14, 15). In this regard, the understanding of chronic pain has evolved from a biomedical perspective to a more holistic approach (16). It is widely accepted that lifestyle factors such as nutrition, sleep, and exercise can significantly impact overall health and chronic conditions, including FS (17). Studies have shown that smokers have a higher risk of developing this condition, suggesting that lifestyle can directly influence its development (18, 19). In fact, previous literature has demonstrated the potential of healthy lifestyle habits to reduce the risk of major chronic diseases (20). However, the adverse effects of smoking, lack of physical activity, and poor diet are reflected in numerous health outcomes (20).

In a previous systematic review, it was determined that chronic pain in general, and shoulder pain in particular, benefit from a multifactorial approach (21). Within this perspective, increasing attention is being paid to dietary factors as potential perpetuators of chronic pain (22). In this context, the World Health Organization (WHO) also acknowledges the importance of diet, stating that “nutrition is emerging as a key modifiable determinant of chronic diseases, with growing scientific evidence supporting the idea that dietary changes have strong (both positive and negative) effects on health throughout life (23).

Modern dietary patterns, characterized by a high intake of ultra-processed foods, refined sugars, and saturated fats, are considered risk factors, and the WHO identifies nutrition as an “important modifiable determinant of chronic diseases” (24). Moreover, diet may play a significant role in inflammation and chronic pain (2). Low intake of nutrients such as omega-3 fatty acids, vitamins B and D, magnesium, and zinc has been associated with neuropathic or inflammatory chronic pain (2). Diets rich in fiber, healthy oils, fruits, and vegetables, and low in sugars and unhealthy fats may help reduce inflammation and disease (25). The lipid profile is a factor associated with primary adhesive capsulitis of the shoulder (26). These lipids, as potent signaling molecules, influence various immune system responses and play a role in the inflammatory processes of several chronic diseases (27). Therefore, a diet rich in anti-inflammatory foods could benefit patients with adhesive capsulitis.

Since the etiology of FS remains largely unknown, it becomes essential to adopt a multidisciplinary approach to address the various factors contributing to the low-grade inflammation generated within the glenohumeral capsule. Recent studies have shown that the metabolic profile of patients directly influences both pain intensity and shoulder functionality. In particular, in an observational study we found a clear relationship between metabolic alterations, specifically decreased levels of liver enzymes (AST, ALT, GGT) and thyroid-stimulating hormone (TSH), which were significantly associated with higher pain levels and reduced shoulder function (28). Furthermore, a recently published meta-analysis confirmed that systemic inflammatory processes related to metabolism may be involved in the pathophysiology of FS (29). These findings highlight the importance of exploring other modifiable lifestyle factors, such as eating habits, given their potential role in regulating chronic inflammation.

In addition, evidence from randomized controlled trials and systematic reviews indicates that structured therapeutic exercise interventions, typically consisting of supervised stretching, mobility, and strengthening programs performed several times per week, are effective in reducing pain and improving range of motion and shoulder function in patients with FS (30–32). These exercise-based interventions, rather than unsupervised or non-structured daily activity, have demonstrated clinically meaningful improvements in pain and functional outcomes. Beyond mechanical and neuromuscular adaptations, exercise may also exert systemic effects through modulation of inflammatory and metabolic pathways (33–35).

Given that certain dietary components exert anti-inflammatory effects while others promote inflammation and may exacerbate or prolong the pathological process (36, 37), this study aims to examine the association between nutritional and lifestyle factors with symptom severity and shoulder function in patients with FS.

## 2 Materials and methods

### 2.1 Study design

The design of the present study is characterized as a cross-sectional observational study involving a group of 57 patients. All procedures were conducted in accordance with the Declaration of Helsinki. Ethical approval and permission to carry out this research were obtained from the Human Research Ethics Committee of the University of Granada (No. 1948/CEIH/2021). In addition, authorization was granted by the National Institute of Health Management of Melilla (Ref: MCC/nmm) to access clinical data from the center for use in this research.

### 2.2 Participants

**FIGURE 1:**
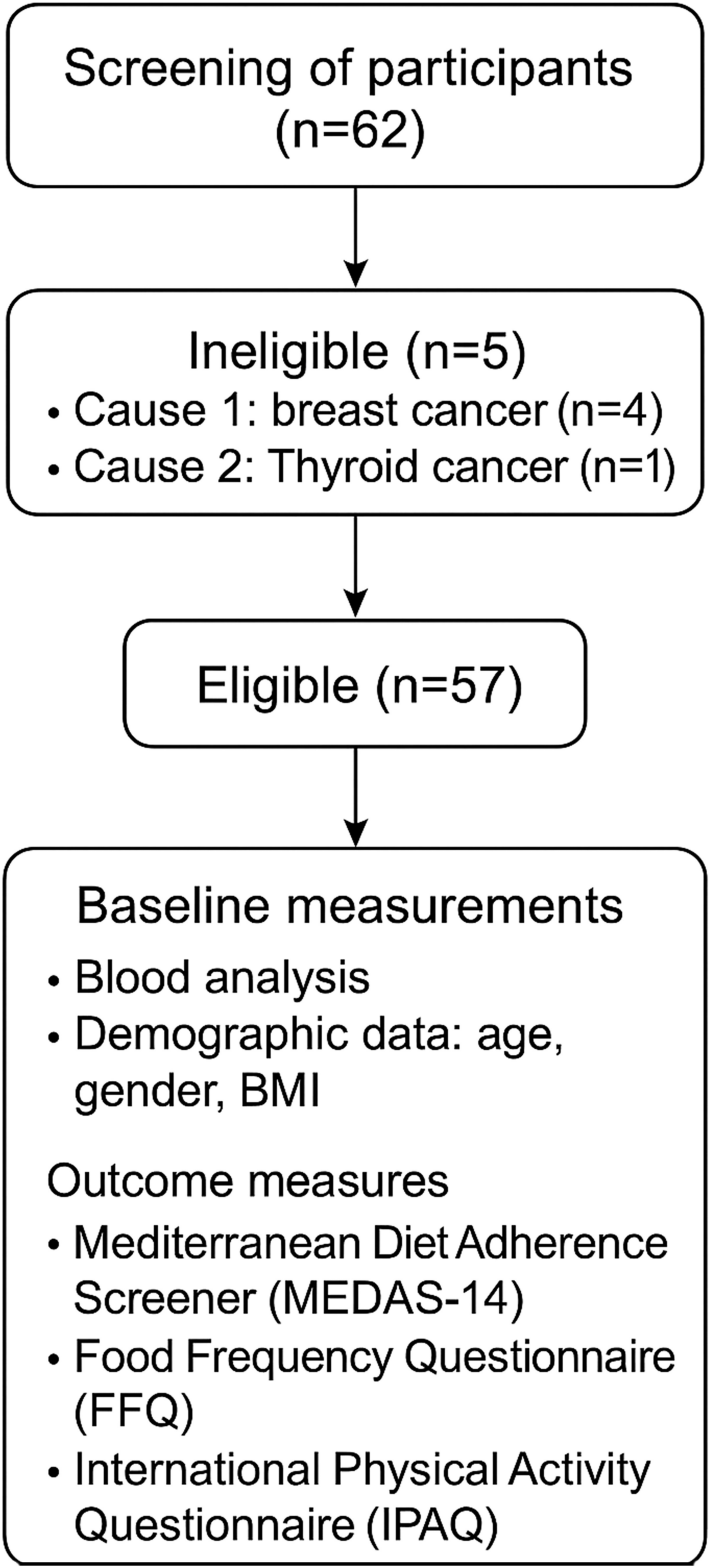
Flow diagram of participant recruitment and selection process.

A total of 57 participants were included in the study, of whom 44 were women and 13 men, after applying the inclusion and exclusion criteria (Figure 1). All participants had a diagnosis of FS and were recruited from the Primary Care Rehabilitation Unit (Central Health Center of Melilla) and the Specialized Care Unit (Melilla Regional Hospital), both part of the National Institute of Health Management of Melilla, Spain.

The participants were not involved in the design, recruitment, execution, reporting, or dissemination plans of our research. The overall results were communicated to the participants via email.

The physiotherapist responsible for recruitment provided participants with information about the study and eligibility criteria. All participants signed an informed consent form to participate in the study. Additionally, the physiotherapist assessed the participants to determine whether they met the inclusion and exclusion criteria.

To protect data confidentiality, each participant was assigned a unique identification number used throughout the study. A list containing these numbers was kept separate from the de-identified data, and statistical analyses were conducted using only the identification numbers. The statistician performing the analyses was unaware of the participants’ identities.

### 2.3 Eligibility criteria

The inclusion criteria were age over 18 years and diagnosis of Adhesive Capsulitis of the shoulder made by a rehabilitation physician. Since there was currently no definitive standard diagnosis for this condition, the diagnosis was based on clinical examination, exclusion of other possible pathologies, and radiological imaging. The clinical diagnostic criteria included: a relevant pattern of capsular tightening, inability to abduct beyond 70°, and limitation of forward flexion above 90° in the affected joint, restriction of passive external rotation by more than 50% of the shoulder joint’s range of motion (ROM), and a clear functional impairment affecting the performance of the patient’s activities of daily living. Participants were excluded if they presented systemic disease (such as rheumatoid arthritis), neurological injury, osteoporosis, hemophilia, or tumors.

### 2.4 Dietary assessment

Dietary assessment was performed using two validated instruments: the 14-item Mediterranean Diet Adherence Screener (MEDAS-14) and a semi-quantitative Food Frequency Questionnaire (FFQ), both originally developed within the PREDIMED study. The MEDAS-14 and the FFQ were administered either self-completed with assistance from trained personnel when necessary.

#### 2.4.1 Adherence to the Mediterranean diet

To assess participants’ adherence to the Mediterranean diet, the MEDAS-14 from the PREDIMED study was employed (38). This tool evaluates the frequency of consumption of key Mediterranean diet components. Briefly, participants received one point for each of the following dietary habits: using olive oil for cooking; consuming at least four tablespoons of olive oil daily; choosing white meat over red meat; eating two or more servings of vegetables per day; consuming at least three pieces of fruit daily; limiting intake of red meat, processed meats (like hamburgers, sausages, or deli meats) to less than one serving per day; drinking fewer than one serving of sugary or carbonated beverages per day; consuming seven or more glasses of wine per week; eating legumes three or more times per week; consuming fish or seafood at least three times per week; eating nuts three or more times weekly; limiting non-homemade baked goods to fewer than two servings per week; preferring poultry (e.g., chicken or turkey) over red meat; and eating two or more meals per week that include sofrito (a sauce made with tomato, garlic, onion, or leeks sautéed in olive oil).

Each item is scored 0 or 1, depending on whether the reported intake aligns with Mediterranean diet recommendations. The total score ranges from 0 to 14, classifying adherence as low (≤ 5 points), moderate (6–8 points), or high (≥ 9 points).

#### 2.4.2 Food Frequency Questionnaire

Dietary intake was assessed using the validated PREDIMED FFQ (39). This semi-quantitative questionnaire includes 137 food items, allowing for the estimation of habitual dietary intake over the previous year. Participants reported their average frequency of consumption for each item, with response options ranging from “never or almost never” to “six or more times per day,” depending on the food.

Standard portion sizes were applied according to the PREDIMED guidelines. To estimate daily nutrient and food group intakes, the frequency of consumption was multiplied by the standard portion size. These values were subsequently matched to the BEDCA database,^1^ a nutrient composition database specifically adapted to the Spanish population.

Total daily energy intake was also estimated, based on the sum of energy contributions from all reported food items, expressed in kilocalories per day.

### 2.5 Shoulder Pain and Disability Index

The SPADI is a questionnaire used to assess pain and disability in patients with shoulder problems. It consists of 13 questions divided into two subscales: pain and disability. The pain questions evaluate intensity during activities or at rest, while the disability questions assess difficulty performing specific tasks with the affected shoulder. Each item is scored from 0 (no pain/disability) to 10 (maximum pain/disability). Scores are summed separately for pain and disability, with higher values indicating greater impairment according to the validated scoring system (40). In the present study, SPADI scores were analyzed as continuous variables, and no clinical cut-offs or categorical classifications were applied.

### 2.6 Physical activity assessment

Physical activity was assessed using the short form of the International Physical Activity Questionnaire (IPAQ-SF), a validated self-report tool that estimated physical activity over the previous seven days by recording the time (in minutes) spent walking, performing moderate and vigorous activities, as well as sitting. Total physical activity was calculated by summing the minutes per week dedicated to walking, moderate, and vigorous activities. Based on these values, participants were classified into three categories: low (< 150 min/week of moderate-to-vigorous activity), moderate (150–299 min/week), and high (≥ 300 min/week), according to current physical activity guidelines [(41); WHO guidelines on physical activity and sedentary behavior: at a glance (42)].

### 2.7 Metabolic profile

Glucose levels and metabolic history, such as diabetes, were obtained from the patient’s medical records and blood tests conducted in the past year at the health center where the study is being carried out. Additionally, values for uric acid, total cholesterol, HDL (High-Density Lipoprotein), LDL (Low-Density Lipoprotein), triglycerides, and C-reactive protein (CRP) were also collected.

### 2.8 Statistical analysis

Data were analyzed by categorizing foods into groups, together with biochemical parameters and nutrient intake. Only nutrients with complete and reliable information available in the BEDCA database for the foods included in the analysis were considered. Nutrients with incomplete or uncertain data quality were excluded to avoid potential bias in the estimation of nutrient intake. Specifically, the following variables were excluded due to incomplete or unreliable information in the database: fructose, sucrose, lactose, detailed fatty acid subtypes (C4 to C22:6 n-3), chloride, beta-carotene equivalents, lutein, and zeaxanthin. Aggregate macronutrient variables such as total carbohydrates (CHOs) and starch were retained when reliable values were available across the food items included in the analysis. This approach was adopted to ensure consistency in the multivariate nutritional analysis and to minimize the influence of missing or poorly characterized nutrient values.

Additionally, two variables describing the carbohydrate-to-fat ratio (CHO/Fat) were included: one numerical and one categorical, classifying values as high or low relative to a reference ratio of 1.5 (60% carbohydrates/40% fats). Models were built including physical activity variables to identify potential confounders. For this purpose, data from the IPAQ-SF questionnaire were used, both, as the calculated categorical scale and as numerical variables representing total minutes, hours, or days of activity. Two complementary statistical approaches were applied. First, a sparse Partial Least Squares (sPLS) analysis was used for dimensionality reduction and to identify associations between multiple predictors and the response variable. For the evaluation of the selected variables, we included the Variable Importance Stability Assessment (VISA), using bootstrap sampling with 3,000 iterations to ensure multiple resamplings of the dataset (43). Model tuning was performed using 5-fold (M-fold) internal cross-validation. Second, an Elastic Net regression model was employed, combining Ridge (L2) and Lasso (L1) penalties to improve predictive performance and interpretability, especially when predictors are numerous and highly correlated. The parameters and code used for the analyses are presented in Supplementary material.

The primary outcome variables were “Total Function” and “Total Pain” scores, where higher values indicate greater disability or more pain. These variables were treated as continuous outcomes in all statistical models, and no clinical cut-offs or categorical classifications were applied. Due to observed sex-related differences, a sensitivity analysis stratified by sex was conducted.

All calculations were performed using the packages mixOmics, and glmnet in R (v4.5.1) (44, 45).

### 2.9 Sample size calculation

Based on previous results from randomized clinical trials and systematic reviews, it was determined that to achieve a statistical power of 90% (β = 0.10) with a significance level of α = 0.05 and an expected standard deviation of 2.0 units on the Numerical Pain Rating Scale (NPRS), at least 22 patients per group are required to detect a clinically relevant difference between subjects. In this study, the final sample consisted of 57 participants, which was considered adequate for the primary planned comparisons (46–48).

### 2.10 Considerations for multivariate penalized modeling

Because no closed-form sample size or statistical power formula exists for sPLS models, sample adequacy in the multivariate context was addressed by limiting model complexity and evaluating robustness through internal validation and resampling procedures. Specifically, sPLS models were optimized using LASSO-based sparsity to retain a restricted number of predictors, thereby reducing overfitting risk. Model tuning was conducted using 5-fold (M-fold) cross-validation. In addition, variable stability was assessed using bootstrap resampling (3,000 iterations), with calculation of VIP and VISA metrics. Bootstrap-based 95% lower confidence intervals are reported in Supplementary Table 3 to allow readers to evaluate estimation precision and stability.

## 3 Results

The sample consisted of 57 patients, of whom 44 were women and 13 were men. The demographic characteristics of the participants are summarized in Table 1. The mean age of the sample was 51.45 ± 10.39 y. Men had a mean age of 47.15 ± 11.39 y, while the women had a mean age of 52.70 ± 9.86.

**TABLE 1:** Socio-demographic data of the sample.

Women								Men						
	Age (y)	Height (cm)	Weight (kg)	BMI	SPADI PAIN	SPADI FUNCT	MEDAS-14	Age (y)	Height (cm)	Weight (kg)	BMI	SPADI PAIN	SPADI FUNC	MEDAS-14
Mean	52.70	166.41	61.57	25.80	40.95	69.91	9.27	47.15	176.58	76.45	24.49	47.42	63.31	11.10
Median	52.00	167.00	61.00	25.68	44.00	71.00	9.00	52.00	177.00	74.40	24.65	44.50	62.00	11.00
Variance	97.36	24.92	98.94	6.19	57.33	114.48	4.07	129.80	16.53	64.50	2.27	41.42	80.90	4.00
SD	9.86	4.99	9.95	2.49	7.57	10.70	2.02	11.39	4.07	8.03	1.51	6.43	8.99	2.00

BMI, Body Mass Index; y, year.

Regarding height and weight, the women had an average height of 166.41 ± 4.99 cm, an average weight of 61.57 ± 9.95 kg, and a mean body mass index (BMI) of 25.80 ± 2.49. In contrast, the men had an average height of 176.58 ± 4.07 cm, an average weight of 76.45 ± 8.03 kg, and a mean BMI of 24.49 ± 1.51.

Concerning pain, assessed using the SPADI questionnaire, women scored a mean of 40.95 ± 7.57 out of 50, while men had a mean score of 47.42 ± 6.43. Regarding functionality, also assessed with the SPADI questionnaire, women recorded a mean score of 69.91 ± 10.70 out of 80, compared to men who obtained a mean of 63.31 ± 8.99 out of 80.

Finally, on the Mediterranean Diet Adherence questionnaire, women scored a mean of 9.27 ± 2.02 out of 14, while men achieved a mean of 11.10 ± 2.00 out of 14 (see Table 1).

In the Elastic Net model including all participants, the following variables were identified as the most relevant predictors, with their corresponding coefficients (see Table 2). No valid Elastic Net model could be constructed for men alone due to data limitations.

**TABLE 2:** Elastic net coefficients from model including all individuals using total pain and total function as predictor.

Total pain		Total function	
	lambda.min		lambda.min
(Intercept)	45.482	(Intercept)	75.934
Thiamine (mg)	–0.305	Manganese (mg)	–2.438
Days walking ≥ 10 sec	–0.132	Thiamine (mg)	–1.466
Decaffeinated coffee (1 cup, 50 g)	–0.090	Vitamin D (ug)	–0.231
Niacin (mg)	–0.065	Iron (mg)	–0.172
Minutes of vigorous physical activity	–0.052	Decaffeinated coffee (1 cup, 50 g)	–0.082
Corn, steamed (82 g)	–0.033	Minutes of vigorous physical activity	–0.069
Minutes of moderate physical activity	–0.008	Monounsaturated fats (g)	–0.062
Iron (mg)	–0.002	Minutes of moderate physical activity	–0.025
Energy (Kcal)	0.000	Total cholesterol (mg/dL)	–0.008
Rice and Pasta	0.001	Carbohydrates (g)	0.000
Non-alcoholic drinks	0.002	Dairy products	0.000
Dairy products	0.002	Tea or infusions (1 cup. 50 g)	0.016
Ice cream (75 g)	0.012	Starch (g)	0.047
Starch (g)	0.024	Garlic (1 clove. 5 g)	0.366
Garlic (1 clove, 5 g)	0.303		

Using sPLS, models were optimized via LASSO to select a limited number of variables. Although two components were retained for visualization purposes, only the first component, which explained the highest proportion of variance, was interpreted. Details of the selected variables, together with VIP, VISA, and bootstrap-based 95% lower confidence intervals, are provided in Supplementary material (Table 3). The confidence intervals were generally narrow for the most influential variables, supporting the stability and precision of the model estimates.

**TABLE 3:** Variables selected by sPLS and their stability metrics (VIP, VISA, and bootstrap 95% confidence intervals).

Variables	VISA	VIPtrue	pval	CI95lower
Glucose (mg/dL)	0.02	0.04	0.96	0.03
Uric acid	0.02	0.49	0.95	0.06
Total cholesterol (mg/dL)	0.50	1.39	0.32	0.70
HDL colesterol (mg/dL)	0.02	0.41	0.96	0.05
LDL colesterol (mg/dL)	0.04	0.77	0.91	0.10
Triglycerides (mg/dL)	0.18	0.93	0.66	0.31
PCR (mg/L)	0.07	0.75	0.85	0.14
Dairy products	0.39	1.20	0.41	0.67
Pudding (one, 140 g)	0.02	0.50	0.95	0.05
Ice cream (75 g)	0.03	0.53	0.94	0.06
Animal protein	0.02	0.19	0.96	0.04
Processed meat	0.04	0.56	0.92	0.07
Fish and seafood	0.03	0.50	0.93	0.05
Vegetables	0.01	0.12	0.98	0.02
Boiled maize (82 g)	0.34	0.90	0.46	0.54
Legumes: lentils, chickpeas, pinto beans or white beans (1 medium serving, 70 g)	0.01	0.04	0.98	0.02
Fruits	0.02	0.33	0.96	0.04
Bread and cereals	0.15	0.90	0.71	0.27
Potato	0.03	0.35	0.94	0.05
Rice and pasta	0.25	1.04	0.56	0.42
Olive oil (10 g)	0.26	1.21	0.56	0.44
Oil and butter	0.01	0.08	0.98	0.03
Bakery	0.02	0.25	0.97	0.03
Alcoholic drinks	0.27	0.74	0.53	0.51
Non-alcoholic drinks	0.31	1.20	0.49	0.54
Water	0.11	0.73	0.77	0.20
Coffee (50 g)	0.35	1.15	0.45	0.56
Decaffeinated coffee (50 g)	1.53	1.69	0.03	1.54
Tea (50 g)	0.27	1.12	0.54	0.48
Ultraprocessed food	0.04	0.73	0.90	0.11
Salt	0.02	0.25	0.96	0.04
Garlic (5 g)	1.50	2.10	0.03	1.62
Sugars	0.03	0.48	0.94	0.05
Total energy	0.07	0.70	0.86	0.14
Proteins	0.85	1.43	0.14	1.09
Fats	0.38	1.35	0.42	0.62
HCO	1.01	1.83	0.10	1.27
Fiber	0.07	0.73	0.85	0.14
Starch_g	1.71	2.14	0.02	1.71
Cholesterol	0.06	0.73	0.88	0.10
Ethanol	0.03	0.37	0.93	0.07
AGM	0.88	1.69	0.13	1.13
AGP	0.01	0.19	0.97	0.03
Glucose (mg/dL)	0.02	0.04	0.96	0.03
AGS	0.07	0.65	0.85	0.15
Potassium	0.05	0.69	0.89	0.11
Sodium	0.01	0.18	0.97	0.04
Calcium	0.05	0.57	0.88	0.11
Phosphorus	0.36	0.88	0.44	0.55
Iron	0.95	1.63	0.11	1.18
Magnesium	0.05	0.68	0.89	0.11
Manganese	0.80	1.46	0.16	1.05
Zinc	0.75	1.25	0.18	1.03
Copper	0.03	0.32	0.93	0.05
Iodine	0.01	0.15	0.98	0.02
Selenium	0.02	0.20	0.95	0.04
Thiamine	1.06	1.74	0.09	1.24
Riboflavin	0.06	0.70	0.86	0.15
Niacin	0.90	1.70	0.13	1.19
Vitamin B6	0.35	1.07	0.44	0.54
Pantothenic acid	0.31	0.97	0.49	0.52
Vitamin B12	0.19	0.90	0.64	0.35
Vitamin C	0.05	0.59	0.89	0.09
Folates	0.24	0.96	0.58	0.41
Vitamin A	0.31	0.84	0.49	0.51
Vitamin D	0.58	1.42	0.26	0.85
How many days a week do you engage in vigorous physical activity (lifting weights, intense activities that leave you breathless, etc.)?	0.47	1.36	0.34	0.73
How long do you engage in these activities each day (in minutes)?	0.58	1.53	0.26	0.85
How many days a week do you engage in moderate physical activity (lifting light weights, dancing, physical activity that does not leave you breathless, etc.)?	0.47	1.17	0.34	0.71
How long each day?	0.89	1.61	0.13	1.12
How many days do you walk for at least 10 minutes?	0.00	0.05	1.00	0.00
How long do you walk each day?	0.02	0.41	0.95	0.05
How much time do you spend sitting during a typical workday? (1: > 4, 2: 4–6, 3: 6–8, 4: < 8)	0.04	0.61	0.92	0.08
Activity level (1: Low, 2: Moderate, 3: High)	0.01	0.34	0.97	0.04

For the full cohort, the most influential variables explained approximately 9% of the variance and included monounsaturated fatty acids, total cholesterol, saturated fatty acids, total fat, starch and carbohydrates, garlic, thiamine, niacin, decaffeinated coffee, iron, vigorous and moderate physical activity variables and the carbohydrate-to-fat ratio (ratio CHO/Fat). Contrary to what was observed in the elastic modeling, a model for men was constructed using sPLS. The first component accounted for 21.3% of the variance, with starch and total cholesterol emerging as key variables.

Overall, both Elastic Net and sPLS approaches yielded consistent findings regarding the dietary variables most strongly associated with total function scores.

**FIGURE 2:**
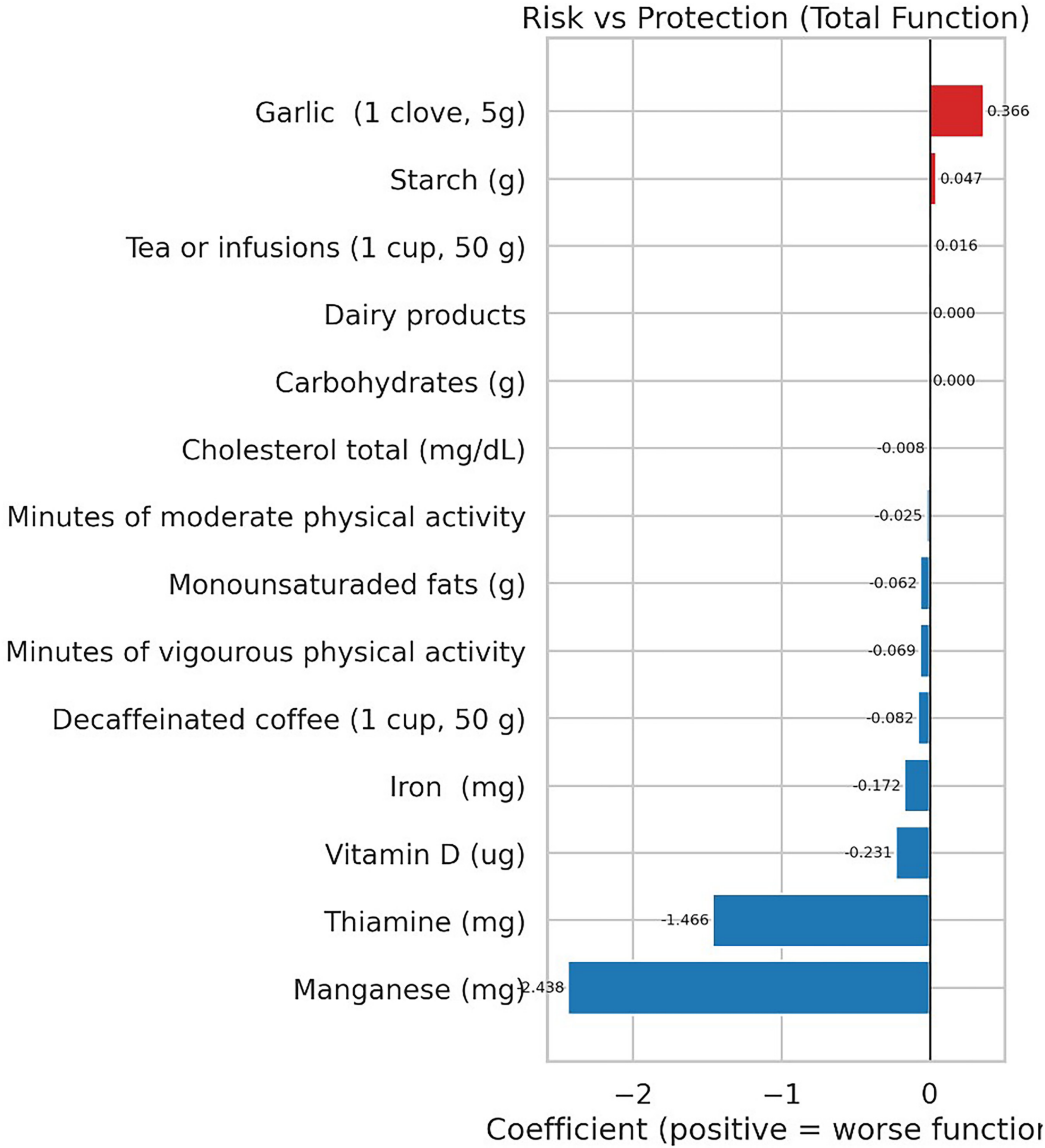
Elastic net coefficients for the full sample, shown separately for total pain (left) and total function (right). Red bars indicate a positive association with worse outcomes (higher pain or poorer function), while blue bars indicate an inverse (protective) association. Bar length reflects the coefficient magnitude at lambda.min.

**FIGURE 3:**
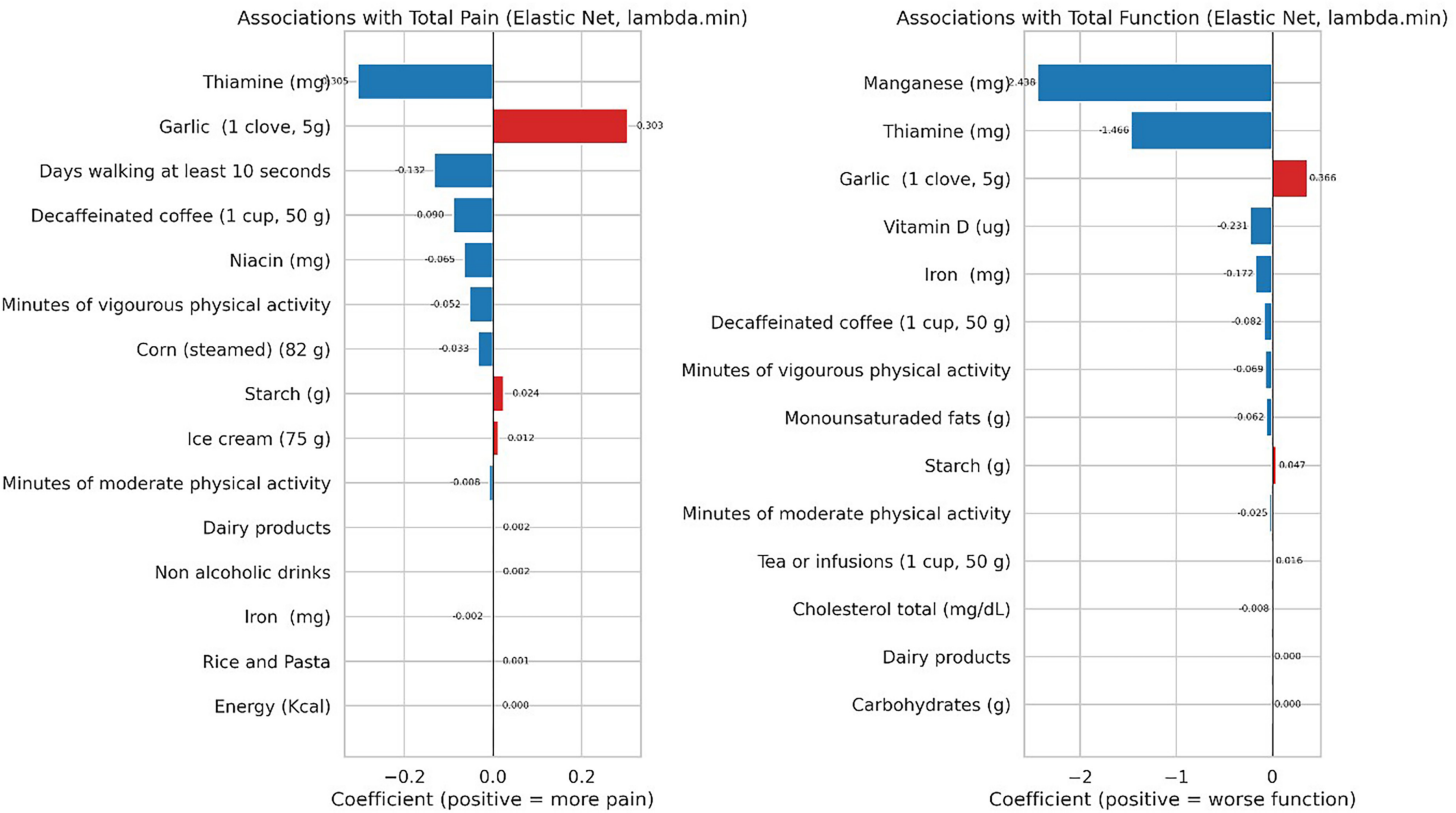
League table of predictors for total function, ranked from most protective (blue, left) to most adverse (red, right), with numeric labels. Negative values indicate better function, while positive values indicate worse function.

**FIGURE 4:**
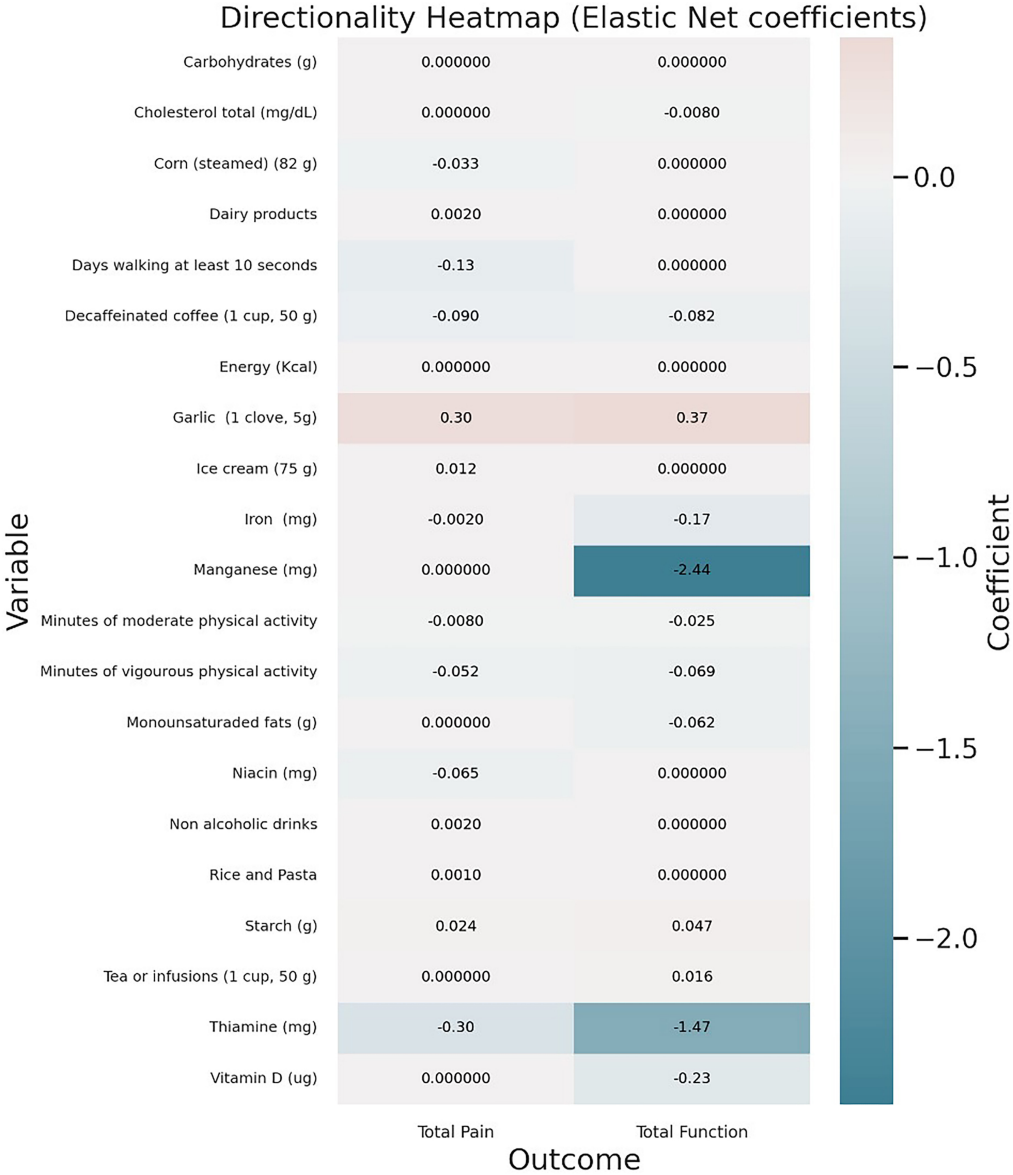
Directionality heatmap with variables in rows and outcomes (total pain and total function) in columns. Both color and numbers indicate the sign and magnitude of coefficients (diverging centered at 0). Blue represents protective associations, red indicates risk associations, and greater intensity reflects larger magnitude.

Figures 2–4 provide a visual representation of the results.

Garlic was positively associated with, both, pain and worse function, whereas thiamine, moderate/vigorous physical activity, decaffeinated coffee, and several micronutrients such as manganese, thiamine, vitamin D and iron showed inverse associations, consistent with less pain and/or better function. On their behalf, manganese and thiamine emerged as leading protective factors, whereas garlic and starch ranked among potential risk-related variables. Other variables show small or near-null effects. Finally, clove appears detrimental in both outcomes (red cells), with starch also linked to poorer function. Micronutrients and physical activity tend to be protective (blue), with stronger effects observed for function.

## 4 Discussion

The results of this cross-sectional observational study suggest that the nutritional profile is associated with functional levels in patients with FS, especially in women, although these associations should be interpreted within the exploratory nature of the study. Through multivariate statistical models (Elastic Net and sPLS), specific nutrients associated with functional impairment were selected as relevant variables within the multivariate models. In the Elastic Net model for the entire cohort, the most relevant variables were starch, CHOs, total cholesterol, garlic, and decaffeinated coffee. Notably, CHOs, starch intake and garlic showed a positive association with worse functional levels, while only decaffeinated coffee showed an inverse association. When analyzed by sex, processed animal products (negative coefficient) and carbohydrates were identified as key predictors of pain and function in women, whereas a valid model could not be constructed for men due to data limitations. Given the small number of male participants, sex-specific findings in men should be interpreted with caution, as they are likely underpowered and exploratory in nature. Accordingly, comparisons between women and men should not be overinterpreted, and male-specific results should be considered hypothesis-generating. The sPLS analysis reinforced these findings. In the total sample, the most influential variables were monounsaturated fatty acids (MUFA), saturated fatty acids (SFA), total fat, starch, CHOs, and total cholesterol, explaining approximately 9% of the variance in functionality. In women, processed animal products alone explained 7% of the variance, while in men the model accounted for 21.3%, with starch and cholesterol emerging as the most relevant variables: However, again, this result should be interpreted cautiously given the small male sample size. Together, both statistical approaches converged in identifying dietary components associated with worse functional outcomes, several of which are commonly described as pro-inflammatory in the literature. Although these associations are compatible with an immunometabolic framework, the absence of direct measurements of circulating inflammatory markers (e.g., CRP, IL-6, TNF-α) or local tissue cytokines limits mechanistic interpretation.

Manganese and thiamine emerged as variables inversely associated with symptom severity, whereas garlic and starch ranked among risk factors. Interestingly, garlic—traditionally described as an anti-inflammatory and antioxidant dietary component—was positively associated with, both, pain and worse functional outcomes in our cohort. This counterintuitive finding warrants cautious interpretation. Rather than suggesting a direct harmful effect of garlic *per se*, the association may reflect residual confounding, reverse causation, or broader dietary context. For example, garlic consumption may be embedded within specific culinary patterns or processed dishes that are themselves rich in refined carbohydrates, saturated fats, or other pro-inflammatory components. Another plausible explanation is that individuals experiencing greater pain or worse health status may intentionally increase their consumption of foods perceived as “anti-inflammatory” or “medicinal,” such as garlic, in an attempt to alleviate symptoms. In observational datasets, this can lead to an apparent positive association between the food and the outcome, even when the food is consumed as a response to the condition rather than a cause of it. This interpretation is consistent with the long-standing traditional use of herbs and spices, including garlic, as health-promoting or therapeutic foods in many dietary cultures (49). Furthermore, the FFQ does not differentiate between preparation methods (raw, cooked, powdered, or included in composite dishes) nor precisely quantify portion sizes, which may introduce exposure heterogeneity. Therefore, this association should be interpreted as exploratory and hypothesis-generating rather than indicative of a causal relationship, and future longitudinal or controlled dietary studies are needed to clarify this finding. Other variables showed small or near-null effects. This ranking provides an exploratory framework to inform future research and hypothesis generation rather than direct clinical prioritization.

Our results are particularly relevant as they align with current literature emphasizing the role of altered metabolic profiles in the pathophysiology of FS (29). A recent systematic review and meta-analysis identified HbA1c as the most consistently elevated metabolic marker in FS patients, suggesting a significant contribution of chronic glycemic dysregulation to the disease’s development (29). Unlike fasting glucose, which did not differ significantly from controls, elevated HbA1c supports the hypothesis that long-term metabolic processes, such as advanced glycation, may induce persistent inflammatory responses in the capsular joint tissues. Furthermore, significantly increased total cholesterol levels have been observed in FS patients (29), reinforcing the role of lipid dysregulation in the development of capsular fibrosis. This finding is consistent with our results, where cholesterol emerged as a significant variable in both statistical models. Such increases may contribute to vascular inflammation and endothelial dysfunction, key drivers in the activation of local pro-fibrotic pathways. Regarding inflammatory markers, the aforementioned meta-analysis highlights a strong association of IL-1β and TNF-α with FS, two well-known cytokines involved in fibroblast activation and dysregulated collagen synthesis (29). Although our study did not directly measure these markers, the association between a pro-inflammatory dietary pattern (high in refined CHOs, starch and processed animal products) and functional impairment may be consistent with mechanisms involving these immune pathways, although these pathways were not directly assessed in the present study.

Clinically, the higher functional disability scores observed in women, along with lower adherence to the Mediterranean diet, may suggest a potential sex-specific association between dietary profiles and symptom manifestation. This phenomenon has previously been reported by Mertens et al. (50), who identified a more prolonged and symptomatic clinical course in women with FS, likely influenced by metabolic and hormonal factors (50).

Other studies have also shown that women with FS tend to have a longer disease duration, more intense pain, and greater functional disability compared to men, highlighting the relevance of these factors in clinical presentation (51). This sexual dimorphism may be linked, first, to hormonal fluctuations related to the menstrual cycle, menopause, and the decline in estrogenic levels, which play a crucial role in regulating inflammation and pain modulation (52). Estrogen have anti-inflammatory and neuroprotective properties; thus, their reduction may promote a chronic pro-inflammatory environment that has been associated with capsular fibrosis and central pain sensitization, although these mechanisms were not directly evaluated in the present study (53).

Additionally, women with FS have been reported to show higher prevalence of insulin resistance, elevated leptin levels, and subclinical thyroid dysfunction—conditions that disrupt immunometabolic homeostasis and promote systemic inflammatory pathway activation (29, 54, 55). These pro-inflammatory effects on macrophages and synovial cells have been associated with chronic inflammatory and fibrotic process in the joint capsule, particularly in women with visceral obesity or metabolic syndrome (4, 56). Moreover, women also present a higher prevalence of affective disorders such as anxiety and depression, which negatively modulate pain perception and are associated with greater central sensitization, potentially explaining the more symptomatic and prolonged course in this group (57, 58). Altogether, the interaction between the female hormonal profile, metabolic dysfunction in pathways such as the HPA axis and the leptin-oxytocin axis, and psychosocial factors may provide an integrative framework to understand the poorer prognosis observed in women with FS, although these multidimensional interactions were not directly assessed in the present study (59, 60). Recent studies have also emphasized the role of metabolic inflammation and immunometabolic dysfunction as key mechanisms in the pathophysiology of FS (60, 61). Specifically, leptin resistance, HPA axis activation, and dysfunction of the GABAergic system have been proposed as mechanisms contributing to capsular fibrosis in FS, with a low-grade inflammatory environment that may be influenced by dietary patterns, although this relationship was not directly evaluated in our dataset (29, 60, 61).

Our findings, which show that higher dietary cholesterol intake was associated with worse functional outcomes in FS patients, align with recent research identifying inflammatory lipoproteins, such as LDL and non-HDL, as independent risk factors for FS (29). These lipoproteins are associated with vascular inflammation and endothelial activation, promoting the expression of adhesion molecules like ICAM-1, which has been found at elevated levels in the joint capsule and synovial fluid of FS patients (62). This vascular activation may be one of the mechanisms linking metabolic alterations and joint inflammation and capsular fibrosis. However, the present study cannot determine whether dietary intake directly contributes to these mechanisms. It has also been reported that the maintenance of a LGI state has been associated with phenotypic changes in joint fibroblasts, contributing to their persistent activation, which in turn has been linked to capsular fibrosis. This chronic fibroblast activation may be influenced by inflammatory mediators derived from pro-inflammatory dietary components (such as refined carbohydrates, starch, and processed animal products), although this pathway was not directly assessed in our study (63, 64). HMGB1, considered a key factor in the perpetuation of FS, is induced by DAMPs and states of cellular stress. Its release facilitates the activation of inflammatory pathways via RAGE and NF-κB, which have been proposed to be associated with dietary patterns rich in oxidized fats or pro-oxidant additives. However, this hypothesis has not been directly explored in our study, and future research should evaluate this potential connection (65). Previous studies have associated the habitual consumption of ultra-processed foods with increased levels of inflammatory markers such as C-reactive protein (CRP), IL-6, and TNF-α, as well as increased intestinal permeability and altered microbiota—factors that contribute to the development of low-grade systemic inflammation (66, 67).

In the context of nutritional factors, dietary components may influence symptom severity in patients with frozen shoulder (68). In our study, higher starch intake in patients with FS was associated with worse function and greater pain. These findings suggest that dietary carbohydrate content may be associated with symptom severity in FS. Accordingly, future longitudinal and interventional studies should evaluate whether dietary patterns lower in refined carbohydrates influence pain and functional outcomes. Previous studies have shown that reducing carbohydrate intake can modulate inflammatory pathways and improve musculoskeletal pain, although such effects have not been specifically demonstrated in FS populations and should therefore be interpreted cautiously in this context (65, 69–71).

The nutritional profile identified in our analysis could represent a dietary manifestation of a broader metabolic and inflammatory phenotype in FS patients. This finding raises the hypothesis that personalized nutritional interventions could potentially influence clinical functionality and metabolic-inflammatory alterations, a possibility that requires confirmation in longitudinal and interventional designs. Future studies should integrate plasma biomarkers with nutritional profiles to validate this hypothesis and identify clinically relevant subtypes with an immunometabolic basis.

Since the multivariate analysis highlighted the role of dietary components such as CHOs, starch, cholesterol, and processed animal products in functional impairment, these may serve as indirect indicators of an unfavorable metabolic environment. In this regard, identifying dietary patterns associated with worse functionality may help inform the design of future nutritional interventions aimed at reducing systemic inflammatory burden. The consistent elevation of HbA1c and total cholesterol levels in FS patients, as reported in previous studies, supports further investigation into whether dietary interventions could influence musculoskeletal function and metabolic parameters. If confirmed in longitudinal and interventional studies, such approaches might be associated with improvements in symptom burden and clinical progression. However, it is important to emphasize that the metabolic data discussed are derived from secondary clinical records and were not collected to establish mechanistic pathways. Therefore, although these findings are compatible with an immunometabolic hypothesis, they do not constitute direct biological evidence that frozen shoulder can be definitively characterized within an immunometabolic model. Rather, they support a conceptual framework that warrants further validation through studies integrating dietary assessment with direct measurement of inflammatory and metabolic biomarkers.

An additional element emerging from our sex-stratified models is the potentially protective association of specific micronutrients, particularly thiamine (vitamin B1) and manganese, in women. Although no causal inference can be drawn from this cross-sectional design and no clinical recommendations can be derived from these findings, these associations are biologically plausible within integrative frameworks that conceptualize frozen shoulder beyond a purely local capsular disorder, incorporating endocrine–metabolic disruption, lifestyle-related low-grade inflammation, endothelial dysfunction, and oxidative stress susceptibility, domains that may be especially relevant in peri- and postmenopausal women (60, 72). Thiamine is essential for mitochondrial energy metabolism and has been linked to redox and inflammatory regulation; inadequate thiamine status has been associated with increased oxidative stress and pro-inflammatory signaling, whereas thiamine-derived compounds (e.g., benfotiamine) have been discussed as potential modulators of oxidative stress-related pathways (73–75). Manganese is a required cofactor for mitochondrial manganese superoxide dismutase (MnSOD), a key antioxidant enzyme involved in mitochondrial reactive oxygen species detoxification and redox homeostasis (76). In the context of estrogen decline, where anti-inflammatory, antifibrotic, and antioxidant buffering may be weakened, such micronutrient-related pathways may hypothetically contribute to biological processes relevant to pain sensitization and tissue repair, although this interpretation remains speculative. Importantly, our data do not demonstrate that increasing intake of these micronutrients improves clinical outcomes. Because we did not measure systemic inflammatory, oxidative stress, or endocrine biomarkers, these mechanistic interpretations remain speculative. Future longitudinal studies integrating dietary assessment with biomarker profiling are needed to determine whether micronutrient intake/status interacts with endocrine-metabolic and immunologic pathways to influence symptom trajectories in women with frozen shoulder (60).

Moreover, in the updated Elastic Net models, several micronutrients emerged as relevant predictors within the model. Thiamine and niacin showed negative associations with pain, which may be consistent with a potential protective role related to their involvement in neuronal metabolism and oxidative stress response (77, 78). Similarly, iron intake showed a modest inverse relationship with pain perception (79). In contrast, foods such as starch, sugary beverages, and ice cream were positively associated with pain, which is consistent with literature describing links between high-glycemic-load diets and systemic inflammation, although inflammatory biomarkers were not assessed in this study (80, 81). Interestingly, garlic classically described as an anti-inflammatory and antioxidant dietary component showed a positive association with both pain and worse functional outcomes in our cohort. This paradoxical finding may be explained by clinical mechanisms: garlic can trigger reflux or heartburn in sensitive individuals (fructans/FODMAPs), which may disrupt sleep, and poor sleep can amplify pain perception (82). Additionally, garlic intake reported in the FFQ is heterogeneous (raw cloves, powder, cooked, or in infusions) (83), with variable doses and bioavailability, potentially diluting true effects and generating spurious associations (84). Further studies are necessary to clarify these relationships, ideally through controlled dietary interventions that account for form, dose, and overall dietary context.

In the sex-specific model for women, additional variables stood out: manganese and vitamin D were inversely associated with both pain and function, which may suggest a potential relevance of antioxidant and anti-inflammatory micronutrients in the female FS phenotype. Meanwhile, foods like ethanol, non-alcoholic sweetened beverages, and ice cream were positively associated with pain, and total cholesterol maintained its negative association with functionality.

Therefore, future clinical trials could consider incorporating well-characterized nutritional intervention arms (e.g., anti-inflammatory, Mediterranean or ketogenic diets) in FS patients, with the aim of assessing their impact on inflammatory markers (IL-1β, TNF-α), lipid profile, chronic glycemia (HbA1c), and joint functionality. A recent randomized controlled trial also demonstrated that an integrative intervention—including physical therapy, exercise, reduction of methylxanthine intake, and restriction of nighttime blue light exposure—was associated with a significant increase in IL-10 levels in FS patients, suggesting the potential relevance of multimodal strategies in modulating systemic inflammation (85). Additionally, the integration of gut microbiome assessments and intestinal permeability biomarkers would provide further insight into the gut-joint axis in FS, particularly given the potential role of ultra-processed foods and dietary additives in epithelial barrier disruption (60).

Finally, characterizing FS patient subgroups according to their diet-metabolic profile may contribute to more refined therapeutic stratification in future research settings, potentially informing personalized approaches beyond conventional orthopedic management.

A particularly relevant line of research involves exploring how the female sex hormone axis, particularly estrogen levels, interacts with nutritional profiles and impacts FS functionality. Certain foods and dietary patterns, such as those rich in phytoestrogens (soy, flaxseed, legumes), healthy fats (omega-3 fatty acids), and antioxidants (berries, cruciferous vegetables), have been associated with beneficial effects in inflammatory conditions, although their specific role in FS remains to be established (86, 87).

In our study, higher consumption of decaffeinated coffee was associated with worse pain and functional outcomes. One plausible explanation is a classic indication bias: patients experiencing more pain or sleep disturbances may switch from regular to decaffeinated coffee, making higher decaffeinated coffee intake a marker of pre-existing worse symptoms. Additionally, decaffeinated coffee is not completely caffeine-free, containing 2–15 mg per cup depending on brand and preparation, which may introduce variability and obscure dose–response relationships. Interestingly, it is also possible that these small amounts of caffeine could exert mild therapeutic effects, suggesting that both mechanisms, the indication bias and low-dose caffeine effects, might contribute to the observed associations.

This research line may contribute to the development of more personalized treatment strategies, integrating nutritional, metabolic, and musculoskeletal perspectives, pending confirmation in prospective and interventional studies.

### 4.1 Strengths and limitations

This study represents, to our knowledge, one of the first exploratory analyses comprehensively examining the nutritional profile of patients with FS using multivariate statistical approaches in relation to clinical functionality and pain outcomes, thus, offering a pioneering Contribution To The Field. The MEDAS-14 and the FFQ were employed as validated tools in both research and clinical practice for identifying healthy dietary patterns and exploring their potential impact on various health conditions. Robust methods such as Elastic Net and sPLS were applied to manage the high dimensionality of nutritional data and minimize overfitting, allowing for more reliable identification of relevant predictors. Notably, our models differentiated variables associated with both function and pain, incorporating sex-specific analyses that revealed meaningful interactions, particularly in women, between dietary components and functional outcomes. The study also proposed well-supported pathophysiological hypotheses, connecting dietary patterns with inflammation, hormonal modulation, and metabolic imbalances. This multidisciplinary and integrative approach, combining nutritional science, immunometabolism, and musculoskeletal pathophysiology, positions diet as a potential area of investigation within integrative management strategies for FS, rather than as an established therapeutic target. Furthermore, the discussion outlines new research directions involving personalized and multimodal interventions, highlighting the originality and clinical relevance of this work while emphasizing its exploratory nature.

However, several limitations should be acknowledged. The unequal sex distribution, with a low proportion of men, limited the ability to construct valid predictive models for the male subgroup, thereby reducing generalizability. Consequently, male-specific findings should be interpreted as underpowered and hypothesis-generating, and direct comparisons between sexes must be approached with caution.

The cross-sectional design precludes inference regarding directionality or causality, and reverse causation cannot be excluded. The relatively small sample size, while adequate for exploratory multivariate analyses, may limit the precision and external validity of the findings.

Additionally, the absence of biological markers prevented direct assessment of inflammatory or metabolic mechanisms underlying the observed associations. Consequently, the proposed immunometabolic interpretation should be understood as a conceptual framework rather than as direct mechanistic evidence.

Another important limitation relates to the use of self-reported dietary assessment tools. Both the FFQ and the MEDAS-14 rely on participants’ recall and subjective reporting of habitual intake over the previous year, which introduces the possibility of recall bias and social desirability bias. Foods perceived as unhealthy may have been under-reported, whereas items considered healthy may have been over-reported. Such misclassification and measurement error are inherent to FFQ-based methodologies and may have attenuated or distorted the observed associations. Although both instruments are validated dietary assessment tools widely used in nutritional epidemiology, and the questionnaires were administered by trained personnel following standardized procedures to improve consistency and minimize misunderstanding, residual reporting bias cannot be excluded.

Finally, although the sample size was adequate for the primary planned comparisons, the relatively modest cohort (n = 57) may limit statistical precision in multivariate modeling. While penalized multivariate approaches such as sPLS and Elastic Net reduce overfitting risk through sparsity constraints and internal validation procedures, some degree of model instability cannot be entirely excluded. To enhance robustness, we implemented 5-fold cross-validation and bootstrap resampling (3,000 iterations), together with stability metrics (VIP and VISA) and confidence interval estimation. Nevertheless, these findings should be interpreted as exploratory and hypothesis-generating, requiring confirmation in larger independent cohorts.

## 5 Conclusion

This study identifies, to our knowledge, one of the first exploratory descriptions of a nutritional and behavioral profile associated with pain and functional status in FS, highlighting specific dietary patterns and micronutrients associated with clinical outcomes. Key findings include associations between higher intake of starch, processed foods, garlic, and sugary products with worse pain and functionality, while micronutrients such as manganese, vitamin D, thiamine, niacin, and iron were inversely related to symptom severity. Decaffeinated coffee was found to be protective in several models, although some sex-specific differences emerged. Physical activity showed consistent benefits across outcomes. These results are consistent with the hypothesis that FS may involve immunometabolic processes influenced by diet, inflammation, oxidative stress, and hormonal balance, particularly in women; however, causal relationships cannot be established based on the present cross-sectional design. Such evidence highlights the importance of considering lifestyle factors within a broader multidisciplinary framework, rather than implying direct therapeutic effects. Integrating personalized nutritional strategies that target pro-inflammatory dietary patterns and promote physical activity represents a promising direction for future research. However, longitudinal and interventional studies are still needed before these approaches can be translated into clinical recommendations. These findings provide hypothesis-generating evidence and support the need for future clinical trials and translational research to further clarify the potential role of immunometabolic nutrition in musculoskeletal disorders.

## Data Availability

The original contributions presented in the study are included in the article/Supplementary material, further inquiries can be directed to the corresponding authors.

## Footnotes

1
https://www.bedca.net/bdpub/
