# Supplementary material for: Impact of nutritional profile on pain and functionality in patients with frozen shoulder: a cross-sectional observational study
Source: Front Med (Lausanne). 2026 Apr 15;13:1785577. doi: 10.3389/fmed.2026.1785577 (PMC13127161; doi:10.3389/fmed.2026.1785577)
Supplement: Supplementary file 1 [file Table_1.docx]

Supplementary Material

# Supplementary Tables

# All statistical models performed in this study are presented in the Supplementary Material, providing detailed results for full transparency and reproducibility.

# Table 1:Elastic Net Females total function

| (Intercept) | 85,9814495 |
| --- | --- |
| Manganese_mg_adj | -5,1928165 |
| VitaminD_ug_adj | -0,5997192 |
| Monounsaturates FAT_g_adj | -0,1622438 |
| How much time on each of those days? | -0,1360509 |
| Olive oil added at the table to salad, bread, and dishes (1 tablespoon, 10g) | -0,1130025 |
| How much time per day? | -0,0755924 |
| Decaf coffe (1 cup, 50g) | -0,0439583 |
| Total cholesterol (mg/dL) | -0,0209564 |
| Calcium_mg_adj | 9,7142E-05 |
| Dairy | 0,00233364 |
| Tear of infusions (1 cup, 50 g) | 0,01136225 |
| Starch_g_adj | 0,02503601 |
| Ice cream (1 cone, cup, or scoop, 75 g) | 0,08389169 |
| Ethanol_g_adj | 0,45262363 |

# Table 2: Elastic Net Females total dolor

| Manganese_mg_adj | -2,2234551 |
| --- | --- |
| VitaminD_ug_adj | -0,3891884 |
| How much time on each of those days? (Min) | -0,1003032 |
| Monounsaturated Fat_g_adj | -0,0957957 |
| Decaf coffe (1 taza, 50 g) | -0,0697793 |
| Boiled corn (small plate or can, 82 g) | -0,03173 |
| How much time per day? | -0,0214063 |
| Total cholesterol (mg/dL) | -0,0118109 |
| Dairy | 0,00083299 |
| Non-alcoholic beverages | 0,00168862 |
| Calcium_mg_adj | 0,00210956 |
| Starch_g_adj | 0,00312253 |
| Tear or infusions (1 cup, 50g) | 0,00551663 |
| Iodide_ug_adj | 0,00705425 |
| Ice cream (1 cone, cu or scoop 75 g) | 0,12084973 |
| Garlic(1 clove, 5g) | 0,23360423 |
| Ethanol_g_adj | 0,27014539 |

# Table 3: Elastic Net ROMFLEX

| Thiamine_mg_adj | 5,32966664 |  |
| --- | --- | --- |
| Iron_mg_adj | 1,11795082 |  |
| Total cholesterol (mg/dL) | 0,14694635 |  |
| How much time do you walk per day? | 0,07330469 |  |
| Boiled corn (small plateo r can, 82g) | 0,06488393 |  |
| Fiber_g_adj | 0,06136606 |  |
| How much time per day? | 0,05350045 |  |
| Custard, flan, pudding (one, 140 g) | -0,0289785 |  |

# Table 4: Elastic Net ROMABD

| Thiamine_mg_adj | 13,24255976 |  |
| --- | --- | --- |
| Iron_mg_adj | 0,924642702 |  |
| Total cholesterol (mg/dL) | 0,197630571 |  |
| Olive oil added at the table to salad, bread, and dishes (1 tablespoon, 10 g) | 0,045342745 |  |
| Legums, lentils, chickpeas, pinto or White beans (1 medium plate, 70 g) | 0,02760513 |  |
| Custard, flan, pudding (one, 140 g) | -0,026833613 |  |

# Table 5:Elastic net ROMADD

| Total cholesterol (mg/dL) | 0,00686077 |  |  |
| --- | --- | --- | --- |

# Table 6: Elastic net ROMINT

| Thiamine_mg_adj | 2,294994221 |  |
| --- | --- | --- |
| Iron_mg_adj | 0,259251931 |  |
| VitaminD_ug_adj | 0,227343285 |  |
| Salt added to dishes at the table (1 pinch from the shaker or between two fingers, 2 g) | 0,215471754 |  |
| Olive oil added at the table to salad, bread, and dishes (1 tablespoon, 10 g) | 0,079477994 |  |
| How much time do you walk per day? | 0,043156 |  |
| How much time per day? | 0,033356501 |  |
| Total cholesterol (mg/dL) | 0,031719919 |  |
| Boiled corn (small plateo r can, 82g) | 0,007709911 |  |
| Niacin_eq_mg_adj | 0,00657268 |  |
| Fiber_g_adj | 0,00349655 |  |
| Water_g_adj | 0,001052535 |  |
| Water | 0,000246322 |  |
| Custard, flan, pudding (one, 140 g) | -0,03198744 |  |
| HDL cholesterol (mg/dL) | -0,036447337 |  |

**
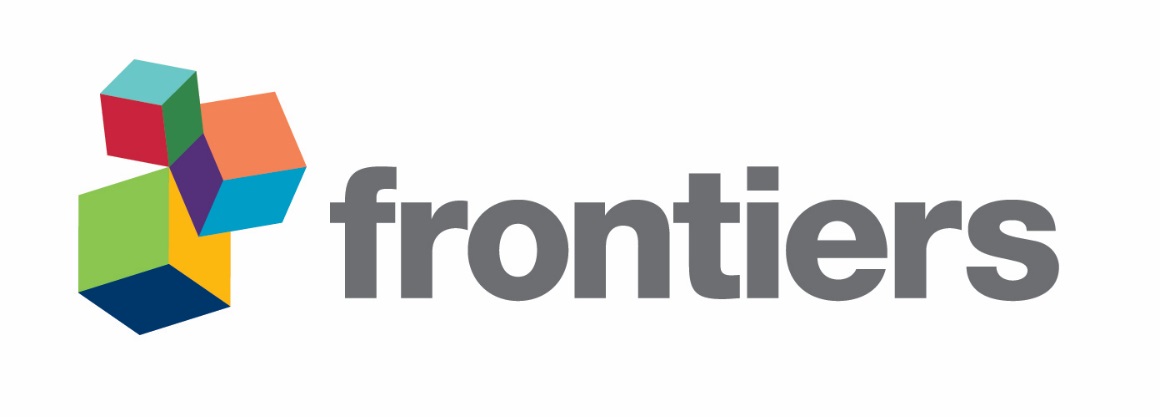
**
